# Coercion, competence, and consent in offenders with personality disorder

**DOI:** 10.1080/1068316X.2015.1109086

**Published:** 2015-11-11

**Authors:** J Zlodre, J Yiend, T Burns, S Fazel

**Affiliations:** a Oxford Health NHS Foundation Trust, Warneford Hospital, Oxford, UK; b Institute of Psychiatry, King’s College London, London, UK; c Department of Psychiatry, University of Oxford, Warneford Hospital, Oxford, UK

**Keywords:** Competence, coercion, personality disorder, informed consent, offenders with mental disorder

## Abstract

Competence to consent to treatment has not previously been examined in a personality disorder cohort without comorbid mental disorder. We examined competence and coercion in 174 individuals diagnosed with severe personality disorder using two validated tools (the MacArthur Competence Assessment Tool for Treatment and the MacArthur Coercion Assessment Scale – Short Form). Competence was not categorically impaired, but there were variations within the sample on dimensional competence measures. Further, there were significant negative correlations between experienced coercion and competence. Higher coercion scores were associated with two components of competence: lower understanding and reasoning. Patients who consented to treatment had higher scores on competence measures and experienced less coercion. These findings suggest that therapeutic approaches that decrease experienced coercion and increase competence may increase the engagement of individuals diagnosed with severe personality disorders in treatment.

## Introduction

Health professionals must seek a person’s informed consent before commencing treatment. Informed consent requires three conditions: adequate information to make a decision, being free of coercion, and competence (also known as capacity) (Appelbaum & Grisso, 1995). Legal coercion may be used to compel some patients to accept treatment when they refuse it. The ethical justifications for coercion are the bioethical principles of *beneficence* when a person is judged to be a risk to himself or herself, or *justice* when a person is judged to be a risk to others.

Competence is usually defined by case or statute law and relates to a specific decision at a particular time, such as accepting medical treatment or being fit to plead in court. In adults there is usually a presumption of competence to make decisions regarding one’s own health and welfare. Assessment of competence is commonly according to the ‘four abilities’ model: understanding information, retaining information, appreciating the significance of the decision and communicating the decision (Grisso, Appelbaum, & Hill-Fotouhi, 1997). Legal systems in most jurisdictions require a categorical, i.e. all or none, judgement regarding competence. Patients who lack competence have the decision made on their behalf if this is necessary. Although categorical decisions are legally required, competence can also be regarded as a dimensional construct (Drane, 1984). For example, decisions that are clearly in a patient’s best interests need a lower level of competence, termed *assent* by Drane (1984), whereas a decision to refuse life-saving treatment requires a higher level of competence that includes *appreciation*, that is a wider perspective on the impact of the person’s decision. Drane suggested that this higher level of competence could be impaired by emotional or cognitive disturbances, including those associated with personality disorders.

Competent individuals making medical treatment decisions should do so voluntarily. Voluntary decision-making is dynamic in that an individual’s perspective regarding his or her freedom to self-determination varies over time and circumstance. Roberts (2002) proposed that ‘voluntarism’ is composed of four domains whose influences in an individual fluctuate: (1) developmental factors; (2) illness-related considerations; (3) psychological issues and cultural and religious values; and (4) external features and pressures. People with personality disorder may be exposed to a variety of factors that affect voluntarism. For example, childhood abuse is a risk factor for developing traits of most personality disorders (Grover et al., 2007) and may impact the development of a person’s sense of agency and autonomy. The *Diagnostic and Statistical Manual of Mental Disorders: DSM-5* (American Psychiatric Association, 2013) defines one of the core components of personality disorder as ‘significant impairments in self (identity or self-direction) and interpersonal (empathy or intimacy) functioning’. The definition of the condition in itself suggests possible deficits in self-direction. In addition particular types of personality disorder, for example antisocial personality disorder with ‘reckless disregard for safety of self or others’, include diagnostic criteria that reflect values that may impact decision-making in the presence of other people. Decision-making is guided and constrained in all people by past experience and values, and it remains questionable whether the values of people with personality disorder should be considered to be an impairment of their voluntary decision-making. Personality disorder is recognized in the psychiatric literature as a mental disorder, although some authors regard it as less empirically valid and based more on values than many other mental disorders (Bendelow, 2010). Therefore, given that voluntarism remains poorly defined and is partly based on values and culture, it requires careful examination, particularly in conditions such as personality disorder, the treatment of which is perhaps more dependent on a values-based and ethical approach.

Voluntarism, as an aspect of informed consent, is particularly important in psychiatric settings, where legal coercion is used routinely, and patients may be suffering from conditions that affect their self-esteem and decision-making. Coercion can be viewed as one of the factors that may reduce voluntarism. Voluntary patients in outpatient consultations may experience greater coercion depending on the interview style of clinicians (Quirk, Chaplin, Lelliott, & Seale, 2012) and voluntarily admitted inpatients can perceive their psychiatric treatment as coercive (Katsakou et al., 2011). Conversely, legally detained patients may regard their admission in a positive way (Bradford, McCann, & Merskey, 1986) and patients who feel their admission to hospital was conducted in a fair and respectful manner may experience less perceived coercion (Lidz et al., 1995). Perceived coercion, as opposed to legal coercion, is associated with poorer subjective clinical outcomes and patient satisfaction (Kallert et al., 2011). It is clear that the subjective experience of coercion, in addition to any legal coercion, influences patients’ experience of treatment. The experience of coercion may impact a person’s decision-making regarding their acceptance of treatment.

High rates of impaired competence to make treatment decisions have been reported in psychiatric patients, for example 60% of psychiatric patients lacked competence to consent to treatment on admission to hospital (Owen et al., 2008), and 29% of psychiatric patients lacked competence during their hospital stay (Okai et al., 2007). Using the MacArthur Competence Assessment Tool – Treatment (MacCAT-T) and dichotomous clinical assessment to examine competence in psychiatric patients, considerable differences have been demonstrated between a range of psychiatric conditions including dementia, schizophrenia, and depression, with the highest rates of impairment in dementia and the lowest in depression (Vollmann, Bauer, Danker-Hopfe, & Helmchen, 2003). Competence to stand trial has been examined by psychiatric diagnosis including personality disorder, with the rate of incompetence at 8%, which although far lower than 66% for psychotic disorder, nevertheless represents a significant minority and is comparable to 13% for mood disorder (Pirelli, Gottdiener, & Zapf, 2011). In a sample of offenders with mental disorder (predominantly psychotic disorder), many were found to be incompetent because they did not appreciate their disorder might increase risk to others (Skipworth, Dawson, & Ellis, 2013). Despite this work, there is little work on competence to consent to clinical treatment in personality disorder. The high prevalence of personality disorder (40%) in psychiatric patients (Newton-Howes et al., 2010), and prisoners (60–70%) (Fazel & Danesh, 2002) indicates that investigation of competence in offenders with personality disorder is necessary.

The current study examined competence and coercion in a cohort of individuals with severe personality disorder who were detained in high-security hospital and prison settings. This represents a selected sample of patients with personality disorder, who are likely to experience high perceived coercion due to the treatment settings. We aimed to investigate the possible links between competence and experienced coercion in patients with personality disorder, and how these may impact on patients’ informed consent. Based on research demonstrating impairments in competence to stand trial in people with personality disorder and impairments in competence in other mental disorders, we hypothesized that a proportion of patients with personality disorder and without other mental disorders would show impaired competence to consent to treatment. In addition, previous work on coercion has shown an association with poorer patient outcomes and therefore we also hypothesized there will be a relationship between experienced coercion, competence to consent to treatment and the decisions of patients to consent to treatment.

## Methods

### Participants

The sample and collection of data have been previously described as part of the ‘Inclusion for *DSPD*: Evaluation, Assessment and Treatment (IDEA) study’ (Burns, Fazel, et al., 2011; Burns, Yiend, et al., 2011). This was an English sample of 174 male prisoners and patients based in high-security prison units and hospitals (34 in Broadmoor Hospital, 38 in Frankland Prison, 37 in Rampton Hospital, and 65 in Whitemoor Prison) selected to participate in the Dangerous and Severe Personality Disorder (DSPD) treatment programme. Inclusion in the DSPD programme required fulfilment of three criteria: (1) more likely than not to commit an offence leading to serious physical/psychological harm according to two separate risk assessment instruments, (2) ‘severe personality disorder’ (defined as Psychopathy Checklist – Revised (PCL-R) score above 30, or PCL-R score above 25 and a personality disorder diagnosis other than antisocial personality disorder, or two or more personality disorder diagnoses other than antisocial personality disorder), and (3) a functional link between personality disorder and offending behaviour (Duggan, 2011). This definition was administrative and created by the UK government. Research assessments at each site occurred within one month of admission or on the anniversary date of admission (where participants had been admitted before the start of the study). Ethical approval was obtained from the South East Multi-Centre Research Ethics Committee in Kent, UK (05/MRE01/94).

The median age of participants was 37.5 years (interquartile range 31.5–43.0), 90% were White, and 46% had completed secondary (high-school) education. All participants had a criminal history, with a median of 12 convictions per participant and median age at first offence of 15 years. Ninety-one per cent (131 of 144) of patients were assessed with the International Personality Disorder Examination (IPDE) (Loranger et al., 1994) and fulfilled criteria for at least one type of personality disorder (median two). The remainder were not assessed with the IPDE, and had a clinically diagnosed personality disorder. The most common diagnoses of personality disorder were antisocial (77%), borderline (45%), paranoid (28%), narcissistic (26%), and avoidant (17%). Almost half of those assessed (61 of 141) had a history of co-morbid axis I disorder (49% of depression or anxiety, and 15% of psychosis) (Burns, Fazel, et al., 2011).

### Measures

A battery of psychometric measures was completed as part of the IDEA study (Burns, Fazel, et al., 2011). These included the MacCAT-T (Grisso et al., 1997) and the MacArthur Admission Experience Survey – Short Form (AES) (Gardner et al., 1993). The results of the Wechsler Adult Intelligence Scale (WAIS) (Wechsler, 1997) and/or the Wechsler Abbreviated Scale of Intelligence (WASI) (Wechsler, 1999) were extracted from the clinical notes if present.

For the purposes of this study, the MacCAT-T interview was adapted to the diagnosis of personality disorder and the proposed treatment. This included an explanation of the diagnosis (the course, impact on behaviour and cognitions, and functional impact), the proposed treatment (group and individual psychological therapy) and the risks and benefits of the treatment. The MacCAT-T was administered by research assistants (RAs) (trainee psychologists), who were instructed in its use by a consultant forensic psychiatrist (SF). The interview included a question at the end as to whether the participant consented to treatment in the DSPD programme. The RAs also made a categorical decision as to the competence of the participant.

The MacCAT-T was designed to enable valid and reliable testing of an individual’s ability to make decisions about the treatment of their mental illness, i.e. their competence to give informed consent. Inter-rater reliability of the MacCAT-T has been demonstrated previously in a sample of psychiatric inpatients with validation by a panel of experts (Cairns et al., 2005). It tests four domains of competence: *understanding* (demonstrated by paraphrasing the information provided), *appreciation* (the ability to apply the abstract information to the person’s own situation), *rational manipulation* or *reasoning* (engaging in a process of rationally weighing up the treatment options), and *expressing a choice* (communicating a decision). Each of these domains is scored individually, *understanding* zero to six, *appreciation* zero to four, *reasoning* zero to eight, and *expressing a choice* zero to two. Higher scores in each domain indicate a greater degree of ability. The MacCAT-T is dimensional, and there is no agreed threshold for competence to make decisions about treatment. As 95% (145/153) of participants had an *expressing a choice* score of two, the highest score, and the rest a score of one, this domain was not examined further.

There were two MacCAT-T interviewers at each site. Twenty-four participants had two separate MacCAT-T interviews by one interviewer separated by approximately one week to examine test–retest reliability within the sample. Eight participants had independent MacCAT-T interviews by two different interviewers and raters to examine inter-rater reliability. Inter-rater and test–retest reliability was evaluated with intra-class correlation coefficients for each domain in a subset of participants chosen randomly.

Inter-rater reliability between RAs was demonstrated with average-measures correlation coefficients (*r*) for *understanding* (*r* = 0.77, df = 7, *p* < .05), *appreciation* (*r* = 0.83, df = 8, *p* < .05), and *reasoning* (*r* = 0.90, df = 7, *p* < .01). Test–retest reliability testing for *understanding* (*r* = 0.40, df = 22, *p* = .11), *appreciation* (*r* = 0.37, df = 22, *p* = .15), and *reasoning* (*r* = 0.47, df = 22, *p* = .08) did not reach statistical significance.

The MacArthur AES measures patients’ experience of coercion (Gardner et al., 1993). There are four scales: *perceived coercion, negative pressures, voice*, and *affective reactions to hospitalization*. The scales are reported separately. Item 10 of the AES was excluded as it addresses the ‘threat of commitment’, and due to the nature of the sample all of the participants were committed. Item 10 forms part of the *negative pressures* scale. Higher scores on *perceived coercion* (range 0–5) indicate less perceived choice and control by the patient regarding the decision to be admitted to hospital. The *negative pressures* scale is reverse marked (range 0–5), so that lower scores indicate a greater perception that others influenced the patient to come into hospital. Higher scores on the *voice* scale (range 0–4) indicate that the patient perceived a greater say in whether they came into hospital. The *affective reactions to hospitalization* scale (range 0–6) examines a range of affective responses (anger, sadness, pleasure, relief, confusion and fright) that can be associated with hospital admission. The distribution of responses to the *perceived coercion* scale was examined for a bimodal distribution as found in the samples that the scale was designed with (Gardner et al., 1993). Cronbach’s *α* was calculated for each of the scales to examine internal consistency. The validity of the AES instrument was examined by comparing scores graphically to the original study (Gardner et al., 1993), and by the calculated correlations between different scales of the instrument. Cronbach’s *α* was 0.79 for the *perceived coercion* scale, 0.67 for *negative pressures*, 0.78 for *voice*, and 0.80 for the negative emotions in the *affective reactions to hospitalization* scale, suggesting good internal consistency.

The WAIS and WASI are validated and reliable instruments used to assess intelligence quotient (IQ), which reflects the verbal and procedural components of cognitive function (Wechsler, 1997). WAIS and WASI results were converted into standardized IQ scores according to normative data provided with these instruments, and internal consistency evaluated. The mean IQ score was 88.8 (*N* = 99, standard deviation 13.8). The IDEA study protocol included previously measured IQ but did not include new IQ assessment, hence the limited sample size.

### Correlation and regression of coercion and competence measures

Data were extracted by site (individually, and prison or hospital) and by axis I diagnosis (mood disorder, psychotic disorder, other axis I disorder, or no axis I disorder). AES scores were imputed by dividing the summed score of each scale by the number of items completed for each scale of the AES. Null scores were excluded. The IQ, MacCAT-T, and AES scores were tested for normal distribution using the Shapiro–Wilk test. Median values and interquartile ranges were reported for each of the scales due to data skewness, and non-parametric statistical tools were used in further analyses.

Correlations between the IQ, MacCAT-T, and AES were examined using two-tailed Spearman’s *ρ* non-parametric coefficient in patients without a history of axis I comorbidity. Differences in MacCAT-T, AES, and IQ scores between those who consented to treatment and those who declined treatment were examined using the Kruskal–Wallis test. Linear regression analysis was used (as dependent variables were continuous) to examine the effect of the AES and IQ on competence as measured by the MacCAT-T scales. Sensitivity analyses were performed to examine the effect of site of treatment and axis I comorbidity on competence and coercion measures by including them as dichotomous variables in the regression.

SPSS Statistics (version 20) was used for all analyses.

## Results

### MacCAT-T and AES scores

From the overall sample of 174 participants, 155 participants completed the MacCAT-T (Table 1). Other participants had refused the interview or were unavailable for interview. The median score for *understanding* was five with interquartile (IQR) range of four to six with 93% of participants having an *understanding* score above three. The median score for *appreciation* was four (IQR 3–4) and 86% of participants had an *appreciation* score above two. The median score for *reasoning* was seven (IQR 6–8) and 89% of participants had a *reasoning* score above four.

Hundred and seventy-one participants completed AES (Table 2). The median score for *perceived coercion* was three (IQR 1–5), for *negative pressures* it was five (IQR 3.75–5), for *voice* two (IQR 1–3), and for *affective reaction to hospitalization* three (IQR 2–4).

### Correlations

Correlations were examined pair-wise between IQ, MacCAT-T, and AES scales (see Table 3) in those without a history of axis I co-morbidity. MacCAT-T scores for *understanding, appreciation*, and *reasoning* correlated negatively with the *perceived coercion* and *voice* subscales and positively with the *negative pressures* subscale suggesting that there is a consistent relationship between the competence and coercion scales. *Understanding* (MacCAT-T) correlated significantly with *perceived coercion* and *negative pressure* (*p* < .01), and *reasoning* with *perceived coercion* (*p* < .01), whereas other parts of the MacCAT-T and AES scales did not correlate at the *p* < .01 level (Table 3). IQ, as a proxy measure of cognitive function, did not correlate with any of the measures. The correlations were also present for a sample which included those patients with axis I comorbidity (data not shown). Thus, there was some support for our second hypothesis that negative correlations between coercion and competence would be found – we found higher coercion scores in some domains were associated with lower scores in certain competence domains.

### Regression analysis

Linear regression, with MacCAT-T *understanding, appreciation* and *reasoning* scores as the dependent variables, showed that *understanding* was not significantly influenced (*R*^2^ = 0.134, *F* (7,73) = 1.458, *p* = .20) by AES scales, IQ, or setting (hospital or prison). The regressions were statistically significant for *appreciation* (*R*^2^ = 0.210, *F* (7,73) = 2.502, *p* < .05) and *reasoning* (*R*^2^ = 0.242, *F* (7,73) = 3.003, *p* < .01). In the *appreciation* model, *voice* (*β* = −0.418, *p* < .01) and hospital site (*β* = 0.266, *p* < .05) were positive predictors of higher scores, whereas for *reasoning* it was prison site that predicted a higher score (*β* = −0.336, *p* < .01). These findings supported our second hypothesis in that patients who felt they had a greater voice in decisions around their treatment were better able to appreciate their condition when making decisions about their treatment.

### Comparison of consenting and non-consenting patients

Twenty-one participants had their MacCAT-T interviews recorded, 11 in hospitals and 10 in prisons (one did not consent to interview and one lacked competence). All of those interviewed were judged by the RAs to have competence to consent to treatment in the DSPD programme. A trained psychiatrist, blinded to MacCAT-T scores, categorically rated the competence status of the 21 participants. The trained psychiatrist listening to the recorded interviews judged that 19 of 20 had competence, with one patient regaining competence between two interviews (at one interview the participant was not able to weigh up the information provided during the competence assessment). This gives a Cohen’s *κ* of 0.9, indicating high agreement between the RAs and the psychiatrist. The RAs assessed all 174 participants as having categorical competence to consent to treatment.

Those participants with competence who had interviews recorded were compared using the MacCAT-T and AES scales according to whether they consented to treatment in the DSPD programme. Eleven consented to treatment and eight did not. Those who consented had significantly higher MacCAT-T scores on *appreciation* and *reasoning*, and higher *negative pressure* and lower *voice* scores on AES. There was no significant difference in *perceived coercion* (AES) or *understanding* (MacCAT-T) scores (see Table 4). These findings supported our second hypothesis, in that consenting patients showed higher dimensional competence and lower experienced coercion.

## Discussion

This study examined 174 offenders with a personality disorder, detained in high-security hospitals or prisons. Their competence to consent to treatment for personality disorder was assessed categorically and using a structured instrument (MacCAT-T). Their subjective experience of coercion was measured using a dimensional scale. The majority of participants were found to be competent and scored highly on a dimensional measure of competence. There were significant negative correlations between the *understanding* and *reasoning* components of competence and measures of experienced coercion. Individuals who consented to treatment had higher competence and lower coercion scale scores than those who did not. These findings suggest that people with personality disorder within secure settings may have high rates of competence to make decisions regarding treatment. Consequently, improving competence and reducing their experience of coercion may make it more likely that they will participate in treatment programmes.

**Figure 1:**
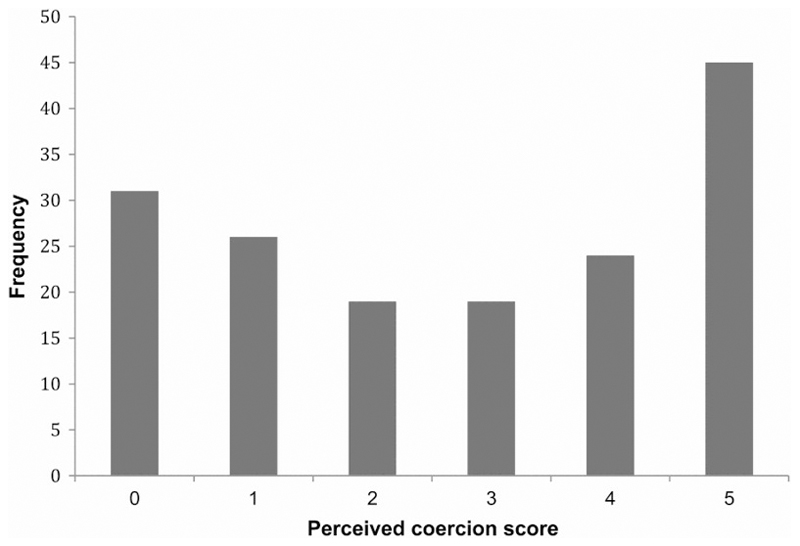
Distribution of *perceived coercion* scores (MacArthur AES) in a severe personality disorder sample (*n* = 171).

This is the first study that, to our knowledge, demonstrates dimensional impairment as measured by the MacCAT-T in a cohort with personality disorder and without axis I co-morbidity, i.e. without any other mental disorder. Our findings suggest that an appropriately adapted MacCAT-T questionnaire can be used in future evaluations of competence in personality disorder. We demonstrated good inter-rater reliability. The lack of significant test–retest reliability could have been due to limited sample size. In relation to the AES coercion scale, the distribution of the *perceived coercion* scores showed less of a bimodal distribution than the original AES sample (Gardner et al., 1993) (Figure 1). This may be due to the high-security environment of the personality disorder sample and suggests external validity of the *perceived coercion* measure. The internal validity of the AES scale is demonstrated by the correlations between the scales, negative in the case of *negative pressures* (see Table 3). While this study had no control group, comparisons can be made with psychiatric patients recruited for other studies. One such study (Grisso et al., 1997) in patients with schizophrenia showed scores in the *understanding* subscale lower than controls and similar to the ones reported in this study. This suggests a degree of impairment in *understanding* in the DSPD cohort. The *appreciation* subscale scores were similar to those of inpatients with depression (Vollmann et al., 2003) and schizophrenia (Grisso et al., 1997), also suggesting impairment. On the other hand, patients in this cohort scored well on *reasoning*.

Why would patients with personality disorder have impaired *understanding* and *appreciation* scores? Understanding requires that information is presented in an appropriate format, that the person has cognitive capacity to retain and process it, and that the person is willing to engage with understanding it. This population has a lower mean IQ compared to the general population, however they are not severely or profoundly cognitively impaired and therefore cognitive capacity was unlikely to impair their ability to understand. The patients lived in restrictive institutions and experienced a high degree of coercion and institutionalization. The quality of relationships with clinicians has been found to impact on patients’ attitudes to treatment and treatment adherence (Day et al., 2005; Sinclair, Willmott, Fitzpatrick, Burns, & Yiend, 2012). Patients may not have engaged with the process of understanding information that was given to them and not sought further information or clarification due to poor relationships with staff. The impairment of *appreciation* on the other hand, could be due to a failure to consider the impact of behaviour outside a highly institutionalized setting or due to the nature of personality disorder. For example, a feature of antisocial personality disorder is not taking responsibility for one’s actions and this could lead to patients not acknowledging the impact of their antisocial behaviour and the potential benefit of treating their condition. Owen et al. (2013) postulated that *understanding* is required for patients to use their *appreciation* and *reasoning*. Thus for patients with a high *understanding* score without competence, those with dementia have impaired *reasoning* (reflecting their cognitive impairment), whereas those with schizophrenia have impaired *appreciation* (reflecting a lack of insight) (Owen et al., 2013). According to this model and our findings, patients with personality disorder may be impaired in competence either because they do not understand the information provided and therefore cannot use the information to reason and appreciate, or can understand the information and reason with it, but fail to appreciate how it is related to their own situation.

Diagnostic criteria for cluster B personality disorders of the DSM-5 (2013) include enduring patterns of behaviour and inner experience that include misinterpreting therapeutic responses as hostile or contemptuous, a disregard for social norms, and impulsive behaviour without consideration of the consequences. People with personality disorder pose considerable therapeutic challenges when informed consent for treatment is sought. For example, the presentation of borderline personality disorder may be characterized by treatment refusal as a way of engaging clinical services (Winburn & Mullen, 2008). The motivation for behaviour associated with personality disorder, such as selfharm, may be unclear, and there may remain considerable disagreement between patient and clinician regarding a formulation of the condition (Szmukler, 2009). Such potential disagreements between patient and clinician regarding the underlying nature of personality disorder may complicate the assessment of competence and the clear determination of informed consent. Furthermore, it has been argued that cluster B personality disorders can be viewed as moral rather than clinical conditions (Charland, 2006), and that treatment of these types of personality disorder must also be based, at least partly, on moral principles. Charland’s (2006) argument pivots on the distinction between clinical and moral treatment, and he does not negate the importance of appropriate clinical treatment in these conditions. As an example of moral treatment, he gives the establishment of a contract at the beginning of treatment with dialectical-behavioural therapy as a way of ‘establishing mutual respect’ and the clinician and patient becoming ‘moral allies’. This type of moral treatment therefore requires a relationship between patient and clinician that is based on a mutual ethical agreement and moral development of the individual. In a recent meta-analysis of treatments for offenders with mental disorder, those that were more voluntary had greater effect sizes, while involuntary ones had no effect or were detrimental (Martin, Dorken, Wamboldt, & Wootten, 2012), suggesting that the mutual acceptance or ‘contract’ of treatment is important. The promotion of competence, voluntarism and informed consent as ways of engaging patients in ethical as well as clinical treatment may enhance the therapeutic relationship and reduce the stigma of mental illness (Winick, 1996). One method may be to design specific interventions to improve therapeutic relationships (Priebe & McCabe, 2008). Measures that decrease perceived coercion are ethically indicated to enable greater voluntarism and may improve patients’ subjective clinical outcomes. Determining the relationships between competence, coercion and consent in personality disorder may allow clinicians and patients to establish a moral framework for treatment, improve agreement regarding diagnosis and treatment, improve clinical outcomes, and decrease the rates of involuntary treatment.

The ethical aspect of treating personality disorder extends into the domain of public protection. Patients with personality disorder are more likely to violently reoffend (Grann, Danesh, & Fazel, 2008) and appropriate interventions to reduce this risk are required. Current treatment approaches in antisocial personality disorder have not demonstrated effectiveness (Wilson, 2014), although there are evidence-based treatments for borderline personality disorder (Leichsenring, Leibing, Kruse, New, & Leweke, 2011). An essential principle in mental health legislation is that offenders have access to treatment for mental illness, and although current treatment approaches for antisocial personality disorder are ineffective, appropriate settings are required to continue to develop potential treatments. The question remains as to the most appropriate setting for such treatments and whether the treatments should be delivered under legal coercion. Public protection and safety of the person are considered grounds for involuntary psychiatric treatment, including that of personality disorder, in the UK (Mental Health Act, 1983). Proposals have been made to alter legislation so that competence to consent to treatment becomes the only criterion for legally coercive treatment (Dawson & Szmukler, 2006). Moving to exclusively competence driven legislation would mean that only those who consent or who lack competence would receive treatment for personality disorder. Due to a high prevalence of competence as demonstrated in this sample, offenders with personality disorder would potentially not be treated if they did not consent or were diverted to prison facilities rather than hospitals (Buchanan, 2010). Our findings suggest that exclusively competence-based mental health legislation would reduce potential access to treatment as a way of protecting public safety. On the other hand, it would mean greater weight would be given to an individuals’ right to self-determination and to moral treatment approaches to conditions whose diagnostic criteria are primarily value-based.

### Limitations

A significant limitation is the lack of a control group, which is a consequence of the original IDEA design, that is a case series. A control group would additionally validate the observed dimensional impairments in competence. Finding a control group appropriately matched to this cohort would be challenging due to the nature of the treatment setting and we instead aimed to draw comparisons with other studies investigating competence. The validity of the DSPD construct as a diagnostic entity has been questioned (Duggan, 2011), and it will comprise more individuals with antisocial traits than general psychiatric or community samples (Yiend, Freestone, Vazquez-Montes, Holland, & Burns, 2013). However, this sample may be most appropriate for study of the interaction of coercion and competence in personality disorder in the context of high-security hospital and prisons. While the findings are specific to the high-security context, the findings may be generalizable to people with personality disorder and would warrant further investigation in other settings. This study is based on the clinical therapy that was offered to patients enrolled in the DSPD treatment programme and does not discuss some of the theoretical issues relevant to the treatment of cluster B personality disorders, including whether individuals with such disorders are more amenable to moral changes in the person rather than clinical therapy. The philosophical approach that seeks to distinguish moral and clinical therapeutic approaches is an important consideration to the treatment of these disorders.

Our findings of positive and negative correlations between parts of the MacCAT-T and AES were statistically significant and in directions to support our hypothesis of an association between higher coercion and lower competence, with no correlations in the opposite direction. However, the correlations were not widely supported by regression analysis. We excluded multicollinearity as an explanation for this lack of effect. There may be confounding factors that explain the correlations observed that were not explored in this study, for example personality factors such as greater external locus of control or the severity of psychiatric symptoms that may influence perception of coercion (Cascardi, Poythress, & Ritterband, 1997). The findings may be due to chance although given the level of statistical significance reached and relationships across several domains, this seems unlikely. The severity of psychiatric symptoms in personality disorder was not examined, and greater severity of symptoms could lead to patients conflating the influence of coercion or detention on treatment decisions. The duration of detention was not examined as a factor that could have influenced the experience of coercion or competence. Future work should investigate these factors in the formation of a model to examine the interplay of these factors in influencing patients’ engagement in treatment of personality disorder. In addition, the relationship between specific personality traits, including psychopathy, and different competence domains and experienced coercion should be examined in future work.

## Conclusion

Patients with personality disorder in a high-security setting, most with antisocial personality disorder, are very likely to have competence to consent to treatment. We found that the MacCAT-T can be used reliably in offenders with personality disorder and there may be dimensional deficits in competence in this cohort. In a smaller sample, 42% of competent patients did not consent to treatment for their personality disorder, and this group experienced more coercion and had lower dimensional competence. Our findings have potential implications for legislators as competence-based mental health laws would potentially prevent offenders with personality disorders accessing treatment in secure hospitals. In addition, our findings suggest that the establishment of mutually agreed frameworks in treatment, such as contracts, may enable patients to engage and consent to treatment and would be foundation of treatment for personality disorder based on ethical and clinical principles.

## Figures and Tables

**Table 1:** Distribution of numbers of participants with different scores on MacCAT-T in cohort of 155 offenders with severe personality disorder.

Score on MacCAT-T subscale	Understanding (%)	Appreciation (%)	Reasoning (%)
7.1–8			64 (42)
6.1–7			25 (16)
5.1–6	86 (56)		33 (21)
4.1–5	33 (21)		15 (10)
3.1–4	26 (17)	108 (70)	8 (5)
2.1–3	7 (5)	25 (16)	6 (4)
1.1–2	2 (1)	12 (8)	3 (2)
0–1	1 (1)	10 (6)	0
Total	155	155	155

**Table 2:** Distribution of numbers of participants with different scores on the MacArthur AES in a cohort of 171 offenders with severe personality disorder.

Score on AES subscale	Perceived coercion (%)	Negative pressures (%)	Voice (%)	Affective reactions to hospitalization (%)
5.1–6				12 (7)
4.1–5	45 (26)	102 (60)		17 (10)
3.1–4	24 (14)	28 (16)	15 (9)	48 (28)
2.1–3	19 (11)	20 (12)	4 (29)	36 (21)
1.1–2	27 (16)	14 (8)	35 (20)	37 (22)
0–1	56 (33)	7 (4)	72 (42)	21 (12)
Total	171	171	171	171

**Table 3:** Correlation (Spearman’s *ρ* coefficient, pair-wise) matrix of IQ, competence, and coercion scales in a cohort of offenders with severe personality disorder.

Instrument or questionnaire	IQ^a^ *ρ* (*N*)	Understanding^b^ *ρ* (*N*)	Appreciation^b^ *ρ* (*N*)	Reasoning^b^ *ρ* (*N*)	Perceived coercion^c^ *ρ* (*N*)	Negative pressures^c^ *ρ* (*N*)	Voice^c^ *ρ* (*N*)	Affective reactions to hospitalization^c^ *ρ* (*N*)
IQ^a^	1.000 (42)	0.041 (38)	−0.007 (38)	−0.094 (38)	−0.042 (42)	−0.185 (42)	0.052 (42)	0.193 (42)
*ρ* (*N*)
Understanding^b^		1.000 (71)	0.411** (71)	0.315** (71)	−0.308** (71)	0.401** (71)	−0.271* (71)	0.023 (71)
*ρ* (*N*)								
Appreciation^b^			1.000 (71)	0.079 (71)	−0.011 (71)	0.155 (71)	−0.021 (71)	0.221 (71)
*ρ* (*N*)								
Reasoning^b^				1.000 (71)	−0.331** (71)	0.212 (71)	−0.204 (71)	0.080 (71)
*ρ* (*N*)								
Perceived coercion^c^					1.000 (79)	−0.729** (79)	0.699** (79)	−0.115 (79)
*ρ* (*N*)								
Negative pressures^c^						1.000 (79)	−0.675** (79)	0.235* (79)
*ρ* (*N*)								
Voice^c^							1.000 (79)	−0.119 (79)
*ρ* (*N*)								
Affective reactions to hospitalization^c^								1.000 (79)
*ρ* (*N*)								

a WAIS and WASI.

b MacCAT-T.

c MacArthur AES.

* *p* < .05 (two-tailed).

** *p* < .01 (two-tailed), and in bold.

**Table 4:** Coercion and competence scores in offenders with severe personality disorder compared by consent to treatment status.

Scale	Median (interquartile range) for all patients (*N* = 19)	Median (interquartile range) for consenting patients (*N* = 11)	Median (interquartile range) for non-consenting patients (*N* = 8)	*p*-Value (Kruskal-Wallis test)
IQ^a^	101 (89–105)	99 (87–109)	104 (102–104)	.30
Understanding^b^	5.8 (4.6–6.0)	5.8 (5.5–6.0)	4.9 (3.1–6.0)	.18
Appreciation^b^	4.0 (3.0–4.0)	4.0 (4.0–4.0)	3.5 (3.0–4.0)	<.05
Reasoning^b^	7.0 (5.0–8.0)	8.0 (7.0–8.0)	5.0 (5.0–6.0)	<.01
Perceived coercion^c^	3.0 (1.0–5.0)	2.0 (0.0–4.0)	4.5 (2.3–5.0)	.08
Negative pressures^c^	5.0 (2.5–5.0)	5.0 (5.0–5.0)	2.5 (1.3–4.7)	<.01
Voice^c^	1.0 (1.0–3.0)	1.0 (0–1.0)	3.0 (2.3–3.0)	<.01
Affective reactions to hospitalization^c^	3.5 (2.0–4.0)	3.5 (2.0–4.0)	3.5 (2.0–4.8)	.75

a WAIS and WASI.

b MacCAT-T.

c MacArthur AES.

## References

[R1] American Psychiatric Association (2013). Diagnostic and statistical manual of mental disorders: DSM-5.

[R2] Appelbaum P, Grisso T (1995). The MacArthur treatment competence study 1. Mental illness and competence to consent to treatement. Law and Human Behavior.

[R3] Bendelow G (2010). Ethical aspects of personality disorders. Current Opinion in Psychiatry.

[R4] Bradford B, McCann S, Merskey H (1986). A survey of involuntary patients’ attitudes towards commitment. Psychiatric Journal of the University of Ottawa.

[R5] Buchanan A (2010). The treatment of mentally disordered offenders under capacity-based mental health legislation. Journal of Mental Health Law.

[R6] Burns T, Fazel S, Fahy T, Fitzpatrick R, Rogers R, Sinclair J, IDEA Group (2011). Dangerous severe personality disordered (DSPD) patients: Characteristics and comparison with other high-risk offenders. International Journal of Forensic Mental Health.

[R7] Burns T, Yiend J, Fahy T, Fitzpatrick R, Rogers R, Fazel S, Sinclair J (2011). Treatments for dangerous severe personality disorder (DSPD). Journal of Forensic Psychiatry & Psychology.

[R8] Cairns R, Maddock C, Buchanan A, David A, Hayward P, Richardson G, Hotopf M (2005). Reliability of mental capacity assessments in psychiatric in-patients. British Journal of Psychiatry.

[R9] Cascardi M, Poythress N, Ritterband L (1997). Stability of psychiatric patients’ perceptions of their admission experience. Journal of Clinical Psychology.

[R10] Charland L (2006). Moral nature of the DSM-IV cluster B personality disorders. Journal of Personality Disorders.

[R11] Dawson J, Szmukler G (2006). Fusion of mental health and incapacity legislation. British Journal of Psychiatry.

[R12] Day J, Bentall R, Roberts C, Randall F, Rogers A, Cattell D, Power C (2005). Attitudes toward antipsychotic medication – The impact of clinical variables and relationships with health professionals. Archives of General Psychiatry.

[R13] Drane J (1984). Competency to give an informed consent – A model for making clinical assessments. Journal of the American Medical Association.

[R14] Duggan C (2011). Dangerous and severe personality disorder. British Journal of Psychiatry.

[R15] Fazel S, Danesh J (2002). Serious mental disorder in 23 000 prisoners: A systematic review of 62 surveys. Lancet.

[R16] Gardner W, Hoge S, Bennett N, Roth L, Lidz C, Monahan J, Mulvey E (1993). 2 scales for measuring patients’ perceptions for coercion during mental hospital admission. Behavioral Sciences & the Law.

[R17] Grann M, Danesh J, Fazel S (2008). The association between psychiatric diagnosis and violent reoffending in adult offenders in the community. BMC Psychiatry.

[R18] Grisso T, Appelbaum P, Hill-Fotouhi C (1997). The MacCAT-T: A clinical tool to assess patients’ capacities to make treatment decisions. Psychiatric Services.

[R19] Grover KE, Carpenter LL, Price LH, Gagne GG, Mello AF, Mello MF, Tyrka AR (2007). The relationship between childhood abuse and adult personality disorder symptoms. Journal of Personality Disorders.

[R20] Kallert T, Katsakou C, Adamowski T, Dembinskas A, Fiorillo A, Kjellin L, Priebe S (2011). Coerced hospital admission and symptom change – A prospective observational multi-centre study. PLoS ONE.

[R21] Katsakou C, Marougka S, Garabette J, Rost F, Yeeles K, Priebe S (2011). Why do some voluntary patients feel coerced into hospitalisation? A mixed-methods study. Psychiatry Research.

[R22] Leichsenring F, Leibing E, Kruse J, New AS, Leweke F (2011). Borderline personality disorder. The Lancet.

[R23] Lidz C, Hoge S, Gardner W, Bennett N, Monahan J, Mulvey E, Roth L (1995). Perceived coercion in mental hospital admission. Pressure and process. Archives of General Psychiatry.

[R24] Loranger A, Sartorius N, Andreoli A, Berger P, Buchheim P, Channabasavanna S, Ferguson B (1994). The international personality disorder examination: The World Health Organization/alcohol, drug abuse, and mental health administration international pilot study of personality disorders. Archives of General Psychiatry.

[R25] Martin M, Dorken S, Wamboldt A, Wootten S (2012). Stopping the revolving door: A meta-analysis on the effectiveness of interventions for criminally involved individuals with major mental disorders. Law and Human Behavior.

[R26] Mental Health Act (1983).

[R27] Newton-Howes G, Tyrer P, Anagnostakis K, Cooper S, Bowden-Jones O, Weaver T (2010). The prevalence of personality disorder, its comorbidity with mental state disorders, and its clinical significance in community mental health teams. Social Psychiatry and Psychiatric Epidemiology.

[R28] Okai D, Owen G, McGuire H, Singh S, Churchill R, Hotopf M (2007). Mental capacity in psychiatric patients. British Journal of Psychiatry.

[R29] Owen G, Richardson G, David A, Szmukler G, Hayward P, Hotopf M (2008). Mental capacity to make decisions on treatment in people admitted to psychiatric hospitals: Cross sectional study. British Medical Journal.

[R30] Owen G, Szmukler G, Richardson G, David AS, Raymont V, Freyenhagen F, Hotopf M (2013). Decision-making capacity for treatment in psychiatric and medical in-patients: Cross-sectional, comparative study. British Journal of Psychiatry.

[R31] Pirelli G, Gottdiener WH, Zapf PA (2011). A meta-analytic review of competency to stand trial research. Psychology Public Policy and Law.

[R32] Priebe S, McCabe R (2008). Therapeutic relationships in psychiatry: The basis of therapy or therapy in itself?. International Review of Psychiatry.

[R33] Quirk A, Chaplin R, Lelliott P, Seale C (2012). How pressure is applied in shared decisions about antipsychotic medication: A conversation analytic study of psychiatric outpatient consultations. Sociology of Health & Illness.

[R34] Roberts LW (2002). Informed consent and the capacity for voluntarism. American Journal of Psychiatry.

[R35] Sinclair J, Willmott L, Fitzpatrick R, Burns T, Yiend J (2012). Patients’ experience of dangerous and severe personality disorder (DSPD) services: A qualitative interview study. British Journal of Psychiatry.

[R36] Skipworth J, Dawson J, Ellis P (2013). Capacity of forensic patients to consent to treatment. Australian and New Zealand Journal of Psychiatry.

[R37] Szmukler G (2009). “Personality disorder” and capacity to make treatment decisions. Journal of Medical Ethics.

[R38] Vollmann J, Bauer A, Danker-Hopfe H, Helmchen H (2003). Competence of mentally ill patients: A comparative empirical study. Psychological Medicine.

[R39] Wechsler D (1997). Wechsler adult intelligence scale – 3rd edition (WAIS-3^®^).

[R40] Wechsler D (1999). Wechsler Abbreviated Scale of Intelligence (WASI).

[R41] Wilson HA (2014). Can antisocial personality disorder be treated? A meta-analysis examining the effectiveness of treatment in reducing recidivism for individuals diagnosed with ASPD. International Journal of Forensic Mental Health.

[R42] Winburn E, Mullen R (2008). Personality disorder and competence to refuse treatment. Journal of Medical Ethics.

[R43] Winick B (1996). The MacArthur treatment competence study: Legal and therapeutic implications. Psychology Public Policy and Law.

[R44] Yiend J, Freestone M, Vazquez-Montes M, Holland J, Burns T, IDEA Group (2013). The clinical profile of high-risk mentally disordered offenders. Social Psychiatry and Psychiatric Epidemiology.

